# Association between Viral Infections and Risk of Autistic Disorder: An Overview

**DOI:** 10.3390/ijerph18062817

**Published:** 2021-03-10

**Authors:** Ahmad Naqib Shuid, Putri Ayu Jayusman, Nazrun Shuid, Juriza Ismail, Norazlin Kamal Nor, Isa Naina Mohamed

**Affiliations:** 1 Advanced Medical and Dental Institute, Universiti Sains Malaysia, Kepala Batas 13200, Malaysia; nqb_ahmad@yahoo.com; 2 Department of Pharmacology, Faculty of Medicine, Universiti Kebangsaan Malaysia, Kuala Lumpur 56000, Malaysia; putri.ayujay@gmail.com; 3 Department of Pharmacology, Faculty of Medicine, Universiti Teknologi MARA, Sg Buloh 47000, Malaysia; 4 Autism Research Group, Department of Pediatrics, Faculty of Medicine, Universiti Kebangsaan Malaysia, Kuala Lumpur 56000, Malaysia; juriza@ppukm.ukm.edu.my (J.I.); norazlyn@ppukm.ukm.edu.my (N.K.N.)

**Keywords:** autism, autism spectrum disorder (ASD), neurodevelopment, perinatal, virus infection, COVID-19

## Abstract

Autism spectrum disorder (ASD) is a neurodevelopmental condition of the central nervous system (CNS) that presents with severe communication problems, impairment of social interactions, and stereotypic behaviours. Emerging studies indicate possible associations between viral infections and neurodegenerative and neurobehavioural conditions including autism. Viral infection during critical periods of early in utero neurodevelopment may lead to increased risk of autism in the offspring. This review is aimed at highlighting the association between viral infections, including viruses similar to COVID-19, and the aetiology of autism. A literature search was conducted using Pubmed, Ovid/Medline, and Google Scholar database. Relevant search terms included “rubella and autism”, “cytomegalovirus and autism”, “influenza virus and autism”, “Zika virus and autism”, “COVID-19 and autism”. Based on the search terms, a total of 141 articles were obtained and studies on infants or children with congenital or perinatal viral infection and autistic behaviour were evaluated. The possible mechanisms by which viral infections could lead to autism include direct teratogenic effects and indirect effects of inflammation or maternal immune activation on the developing brain. Brain imaging studies have shown that the ensuing immune response from these viral infections could lead to disruption of the development of brain regions and structures. Hence, long-term follow up is necessary for infants whose mothers report an inflammatory event due to viral infection at any time during pregnancy to monitor for signs of autism. Research into the role of viral infection in the development of ASD may be one avenue of improving ASD outcomes in the future. Early screening and diagnosis to detect, and maybe even prevent ASD are essential to reduce the burden of this condition.

## 1. Introduction

Autism spectrum disorder (ASD) is a neurodevelopmental disability characterised by social and communication impairment and by restricted interest, repetitive and stereotyped behaviours [1]. The degree of developmental disabilities among individuals with ASD is variable, but the impacts on affected individuals and their families are life-changing [2]. The earlier studies of autism prevalence published in the 1960s and 1970s reported approximately 4 to 5 cases per 10,000 children [3]. Since the 1990s, a substantial increase in the estimated prevalence of autism in the United State (US) have been reported. ASD is one of the most prevalent neurodevelopmental disorders that affect children today. The number of children with autism are projected to exceed the number of children with cancer, juvenile diabetes, and paediatric acquired immune deficiency syndrome (AIDS) combined [4]. Among all mental disorders that commence in childhood, ASD was the leading cause of disability in children under five years of age. In 2010, approximately 52 million cases of ASD were reported around the world with the prevalence of 7.6 per 1000 or 1 in 132 persons [5]. The Autism and Developmental Disabilities Monitoring (ADDM) Network in the US estimated the prevalence of ASD among eight years children in 2014 was 1 in 54; which has increased from 1 in 150 in year 2000 [6]. The increase of ASD prevalence has been attributed to increase in public awareness, changing diagnostic standards, earlier diagnosis of autism, and development of treatment target.

Frustratingly little is understood about the causal mechanism underlying this complex disorder, but it is known that ASD is strongly influenced by genetic and environmental factors. It is also increasingly clear that ASD is a heterogenous disorder. In addition to complex genetic factors, environmental exposures during critical periods of early neurodevelopment is associated with autism [2]. Prenatal environment, including maternal infection and inflammation, as well as perinatal and postnatal exposures to various substances are increasingly recognised as potential risk factors for autism [7]. Genetic background could also influence an individual’s susceptibility to infection [8]. Since the association of autism with congenital rubella infections was noted more than 50 years ago, numerous other infections have been connected to the incidence of autism in the intervening years [9]. Epidemiological studies and case reports demonstrated the link between exposure of viruses such as rubella [10,11], measles and mumps [11], polyomaviruses [12], cytomegalovirus [13,14], and influenza [11,15] to the risk of autism. Animal studies have also indicated that prenatal or early postnatal infections could result in both acute and persistent neurological and behavioural abnormalities in the offspring, displaying features reminiscent of autism and schizophrenia [16,17]. For instance, respiratory infection with human influenza virus in pregnant mice at mid-gestation resulted in behavioural and pharmacological abnormalities thought to be due to maternal antiviral immune response that consequently affected foetal brain development [15,18].

The potential role of immune dysregulation and autoimmunity in autism has gained particular traction given that inflammation, cytokine dysregulation and anti-brain antibodies could have significant impact on brain development [9]. Immune dysfunction is in fact a viable risk factor contributing to the neurodevelopmental deficits observed in ASD [9]. It has been postulated that viral infection may lead to ASD through direct infection of the CNS, through infection elsewhere in the body that could trigger diseases in the CNS or through the alteration of maternal or offspring immune response [8]. Many previous studies have implicated various viral infections with ASD, although some other studies have reported no association. In this review, we summarise the growing body of evidence, encompassing human studies and experimental animal studies that supports a possible connection between viral infection and the risk of autism.

## 2. Method

An electronic literature search for relevant articles in this narrative review was performed using Pubmed, Ovid/Medline, and Google Scholar database. Five types of viruses, namely rubella, cytomegalovirus, influenza virus, Zika virus, and COVID-19 were considered for inclusion owing to their strong association and possible risk to ASD. The following search terms were used in the search process: (1) Rubella and autism, (2) Cytomegalovirus and autism, (3) Influenza virus and autism, (4) Zika virus and autism, and (5) COVID-19 and autism. A total of 141 articles were retrieved on the basis of the search terms for this review. Studies on infants or children with congenital or perinatal viral infection and autistic behaviour including case-control, epidemiological, and cohort studies were evaluated. Studies that report no association between viral infection and autism were also included. No date limit was applied but studies published in English language specific to viral infection and the risks of ASD were taken into consideration.

## 3. Viral Infection during Pregnancy

In response to an infection, a variety of cells of both innate and adaptive immune systems become activated and participate in the effort to control and eliminate the invading pathogens. Viruses can affect neurons by killing them directly through cell lyses or by inducing apoptosis. The activation of innate and adaptive immune responses could also eventually lead to neuronal damage through direct damage, killing, release of free radicals, cellular activation and inflammation [19,20]. A substantial number of published evidence and reports have suggested that neurobehavioural diseases such as ASD could be caused by systemic and neuropathic viral infection. Neurodegenerative and neurobehavioural diseases may result in chronic and progressive loss of the structure and functions of neurons in the CNS [21,22].

Women become more susceptible to infections during pregnancy. Several mechanical and pathophysiological changes occur and immune adaptations are required to shelter the foetus [23]. Pregnant women also may be more severely affected by infections as compared to non-pregnant women. Viral infections that occur during pregnancy may or may not manifest clinical signs in the mother. They may exhibit no effect, indirect effects or direct effects on the foetuses [24]. Viruses that crossed the placental barrier to reach the foetus could cause devastating effects on foetal development [25]. Foetuses and infants are at high risk of viral damage as their CNS are not fully developed at birth or still immature after birth. Maternal viral infection during pregnancy result in an adverse intrauterine environment and increases the risks of neuropsychiatric disorders such as ASD significantly [26]. It has been hypothesised that viruses might act as teratogens contributing to autism, given that the teratogenic effects of prenatal infections such as rubella and cytomegalovirus on the CNS are well established [27]. A large Swedish cohort study found that the risk of ASD was elevated in pregnancies affected with a broad spectrum of infectious agents in all trimesters of pregnancy. The study also reported consistent associations of inpatient diagnosis of infection and ASD with comorbid intellectual disability [28].

Neuroinflammation during early foetal development may induce psychopathological and neuropathological features of ASD. Indeed, neuroimmune factors play an important role in the aetiology of neurological and neuropsychiatric disorders including those with early pathogenic onset of brain development [29]. The foetal inflammatory response to intrauterine infection in particular appear to contribute to neonatal brain injury and subsequent neurological disability [30]. The T-helper cells, also known as CD4 T-helper cells are a type of T-cells that play a central role in the adaptive immune system through their capacity to help B-cells make antibodies, to induce macrophages and to recruit neutrophils, eosinophils and basophils to sites of infection and inflammation, and, through their production of cytokines and chemokines [31]. T-helper-17 cells in particular are responsible for immune response against an extracellular challenge and their dysregulation is thought to underlie numerous inflammatory diseases, and have been suggested to have a role in ASD [32]. Upon viral infection, the production and release of various cytokines substantially affects the immune and CNS cells through receptor-mediated events [8]. Cytokines can be produced directly by the brain or reach the CNS by crossing the immature blood–brain barrier (BBB). Interleukins (ILs) are a group of cytokines released in response to various inflammatory processes and they are the logical candidates for disruption of foetal brain development [33]. The dysregulation of inflammatory cytokines such as IL-1, IL-6, and IL-17, and the immunomodulator cytokines such as IL-2 has been found in whole blood samples of ASD patients, which highlight the importance of immune response in the development of ASD [34].

Notably, IL-6 has been considered as an indicator of maternal systemic inflammation that could influence placental–foetal interactions and subsequent foetal brain development, thus increasing the risk of neuropsychiatric disorders in the offspring [35]. It may lead to cognitive and behavioural deficits by altering the formation of synapse and affecting synaptic function in the affected offspring [36]. The impairment of normative synaptic signalling and transmission may alter the balance of neurotransmitters and the number of excitatory versus inhibitory connections in the developing brain. These events potentially set the stage for a wide range of adverse developmental outcomes [35]. As evidence in a longitudinal study, the increase levels of IL-6 during pregnancy could alter brain architecture, executive function and working memory abilities in neonates [35]. As inflammatory markers particularly IL-6 are expressed throughout the brain, it appears that cytokines have the potential to affect normative growth processes at every stage of foetal brain development.

A number of studies have demonstrated the presence of an inflammatory-like state in the brain, cerebrospinal fluid (CSF) and peripheral immune system of many ASD samples [37,38,39]. An inflammatory-like state was found in brain post-mortem of person with autism as indicated by activated cytokines and activated microglia and astrocytes [37]. Cytokine elevation was also found in the CSF of children living with autism indicating that the immune-activated state is established early and appears to be permanent. Abnormalities in the peripheral immune system are also possibly related to the inflammatory-like state in the CNS [40]. Several cytokines and chemokines such as IL-1β, IL-6, IL-8, IL-12p40, and IL-17 were elevated in the plasma of very young children with ASD, and these findings were associated with communication impairment and aberrant behaviours [41,42,43]. Briefly, the physiopathology of ASD involves several modifications at the genetic and immune levels such as elevation of inflammatory cytokines and abnormal immune response. Based on the relationship between the inflammatory cytokine response due to viral infection in pregnancy and brain injury, it was believed that maternal cytokines dictated the type and severity of immune response and this could anticipate brain pathology [25]. Various evidence supported the theory that maternal infection and/or inflammation occurring during the critical period of foetal development impair brain structure and function, and could potentially alter cognitive and psychological function later in life [25].

As a whole, the combination of genetic susceptibility and maternal infections increases the likelihood of behavioural deficit in the offspring, depending on the intensity of infection and the gestational age (early vs. late and as late as the lactation stage) [44]. Maternal immune activation-associated abnormalities have been described in vitro and in animal studies, post-mortem data and genetic studies in multiple brain cell types, all of which were implicated across neurodevelopmental disorders [45]. In brief, viral infection leads to the release of pro-inflammatory cytokines and activation of T-helper-17 cells in the maternal blood circulation [32,46]. Animals models of maternal immune activation show that maternal inflammatory response could affect the early programming of different behaviours including the ability to socialise and communicate, and the regulation of stereotypic behaviours. These effects were expected to be similar to the effects on the human brain [47]. All of the studies over the years have presented evidence both for and against the association of autism with various viral infections.

## 4. Specific Viral Infections and Association with ASD

### 4.1. Rubella and ASD

Rubella virus which is an enveloped RNA virus is a member of Togoviridae family. It has been recognised as a disease for approximately 200 years and humans are the only natural reservoir for rubella virus. The virus can be found in nasopharyngeal secretion, blood, faeces, and urine of the patients with clinical signs, but patients with subclinical diseases are also infectious [48]. It can be transmitted through respiratory droplets that are exhaled, sneezed, or coughed by infected individual, and the virus then spreads from the infected cells of upper respiratory tract to the vascular system. The virus replicates in lymphoid tissue of the nasopharynx leading to multiple organ systems infection. Viraemia occurs 5 to 7 days following respiratory transmission and replication of virus in the nasopharynx and regional lymph nodes, spreading throughout the body [49].

Rubella is a systemic viral infection that usually causes benign illness resembling a mild case of measles. However it can cause severe birth defects known as the congenital rubella syndrome (CRS) when infection occurs early in pregnancy [49]. CRS includes various defect including deafness, cataract, encephalitis, heart abnormalities and intellectual disabilities [50,51]. Rubella virus is one of the infectious agents that can infect the foetus prenatally. When the infection occurs in the first trimester of pregnancy, the rates of foetal infection can go up to 50%, while the pregnancy outcomes include spontaneous abortion, foetal infection, stillbirth or foetal growth restriction and CRS [52]. More than 20,000 children were born with CRS after an outbreak of over 12.5 million cases of rubella from 1963 to 1965 [53]. Congenital rubella virus infection was first linked to defects in the newborn by Gregg [54] during a major rubella epidemic in Australia in 1940. In the 1970s, Stella Chess found that children with congenital rubella had higher incidence of autism, 200 times than that of general population at that time [55,56]. High prevalence of autism in children with CRS during the rubella epidemic in 1960s built the theory that maternal infection with immune system activation in pregnancy led to autism in the offspring [55,56].

The association between rubella and autism was made based on population studies and case reports. As reported in a study on the behavioural consequences of congenital rubella by Chess et al. [10], ASD is one of the many outcomes associated with CRS. Congenital viral infections of the CNS could produce more severe and complex symptoms of autism [56]. In the study, children with CRS were re-examined at age 8 to 9. It was shown that 7.4% fell into the category of autism which was higher than the estimated prevalence in the general population at that time. Based on Kanner’s classical criteria, from the total of 741 children with autism per 10,000 children having congenital rubella, 412 had complete syndrome of autism and 329 had partial syndrome. In an earlier study, rubella vaccine challenge was used in attempt to retrospectively diagnose prenatal rubella in children with autism. It has been found that autistic children had an altered immune response to rubella vaccination indicating that they had been congenitally infected with the rubella virus [57].

The clinical features of CRS can be generally classified as transient, self-limiting or permanent. However some of the CRS features may not present until adolescence or adulthood and these are referred to as “late manifestation” or “delayed manifestation”, which include auditory and visual disorders, cardiac disorders, endocrinal disorders, oesophageal and gastrointestinal problems, and urogenital problems [58,59]. Hwang and Chen [60] reported a 20-year-old male had been treated with an antipsychotic and antidepressants since the age of 12 due to unstable moods, violent and stereotypic behaviours. He was diagnosed to have CRS based on the history of maternal rubella infection during gestation and his multiple congenital physical defects. This highlighted the importance of careful survey and evaluation of late manifestation of CRS. The exact way in which rubella continues to affect an individual with CRS is still unknown but the experts believed that it can be due to a persistent infection of rubella virus in the affected organs, or autoimmune responses to old infection [61,62]. In another case reports, young males with congenital rubella who were diagnosed with autistic disorder, also suffered from visual and hearing impairments [63] and bipolar disorder [64]. Sensory impairments and developmental delay are common in children with congenital rubella. Recently, Toizumi, et al. [65] published a study on developmental difficulties and sensory defects in children with CRS following a large rubella outbreak in Vietnam in 2010. Developmental difficulties combined with sensory dysfunction were suspected in 95% of the children with CRS, while 41% of them were suspected to have autism after 3 to 5 years of the outbreak.

The signs and symptoms of rubella infection has been proposed to be associated with retinoid toxicity that occur due to the alterations in the maternal hepatic metabolism of vitamin A [49]. It is hypothesised that rubella infection impairs the enzyme responsible for the conversion of retinol to retinoic acid and the catabolism of retinoic acid. Consequently, the accumulation of vitamin A compounds, collectively termed as retinoid, results in liver damage and dysfunction. The impairment of hepatic mobilisation and secretion of the carrier protein retinol-binding protein caused serum retinol to decline and stored retinoids compound from the dysfunctional liver to enter the circulation, thus inducing the clinical features of rubella. In the early weeks of pregnancy when fetal development is strongly regulated by endogenous retinoids [66], such damage to the maternal liver may result in the entry of retinoid compounds into fetus. Retinoid toxicity may interrupt normal fetal development, causing dysmorphogenesis, brain damage and autism [49]. Hence it is suggested that the risk of ASD and related neurodevelopmental disorders are associated with virus-induced changes in retinoid metabolism and hepatic dysfunction. Together, they are responsible for the malformations and damage to many organs including the brain, as seen in the early and late manifestation of CRS.

CRS was almost eradicated in the developed world after widespread vaccination. According to the Centers for Disease Control and Prevention (CDC), rubella and CRS were declared eliminated in the US in 2004 and in 2015 the Pan America Health Organization of the World Health Organisation (WHO) announced that the Americas region is the world’s first region to eliminate rubella and CRS. From 2001 to 2010, rubella vaccination had prevented hundreds and perhaps thousands of ASD cases in the US by preventing CRS [67]. However, in the recent appraisal, Hutton postulated that rubella might still cause autism even in vaccinated population [68]. Rubella has not been eradicated and is still common in other parts of the world. For instance, in 2018 there was an outbreak of rubella in susceptible children in Ethiopia, Africa who were unvaccinated against rubella [69]. Globally, rubella was serologically detected in up to 5% of pregnant women. Though vaccination is essential, some women may not respond to the vaccine and the antibody titers may decline over time, leaving older mothers more at risk. Based on the current evidences, research must be continued to determine the role of rubella in ASD [68].

### 4.2. Cytomegalovirus and ASD

Human cytomegalovirus (CMV) is the most complex member of the human herpesvirus family and is highly species specific; humans are the only hosts of human CMV [70]. This virus belongs to the neurotropic beta-herpes virus family and can infect various cell types including epithelial cells, endothelial cells, smooth muscle cells, neurocytes and sustentacular cells of the CNS. Histopathologic and immunohistochemical examinations of necropsy tissues demonstrate that the virus enters via the epithelium of the upper alimentary, respiratory or genitourinary tracts. It is excreted in the body fluids such as urine, saliva, tears, semen, milk and cervical secretion for months to years and the virus can appear following primary infection, reinfection or reactivation. Infants can be infected following transmission from their mothers via the placenta, during delivery or by breastfeeding. Ingestion of genital secretion or breastmilk of infected maternal is the main perinatal route of infection [71]. CMV can invade the CNS during any stage of development and maturity of the nervous system, resulting in congenital or perinatal infection. The invasion of CMV into the CNS can cause neurodevelopmental disorders or other neurologic diseases through the interference of neural stem cells proliferation and differentiation [72].

Little is known about the pathogenesis of CMV infection and its associated damage in the foetal CNS [72]. Unlike influenza, CMV infection are asymptomatic and do not result in hospitalisation, but despite being sub-clinical, this virus still induces an immune response [73,74] that could lead to the occurrence of neurodevelopmental disorder. Cannon and Davis [75] reported that congenital CMV infection was the major cause of birth defects and childhood disorders in the US. Approximately 40,000 babies (0.2 to 2% of all deliveries) were born with CMV, with 400 fatal cases reported each year, leaving 8000 children with permanent disabilities such as hearing and vision loss, or intellectual disability [75]. Only 10–15% of the children exhibit clinical signs at birth but despite appearing asymptomatic at birth, they are at risk for developmental sequelae [76]. In addition, CMV can be reactivated throughout lifetime of the host. This occurs when the latent virus switches to a lytic phase of replication when triggered by a combination of external and/or internal cellular stimuli [77]. Not much is understood on how the reactivation of the virus could affect maternal immune system and what consequences they might have on foetal neurodevelopment and ASD [78]. It was postulated that chronic maternal immune activation due to persistent viral reactivation may be triggered by pregnancy-related stress and could adversely affect foetal brain development.

Herpesviruses can induce inflammation primarily by blocking the interferon that inhibits the host’s antiviral defence. There is evidence that CMV infection affects CD8 T-cell function [79] and maternal CD8 T-cell trafficking in placenta play a role in mediating perinatal brain injury [80]. CD8 T-cells are mediators of adaptive immunity that comprise of both cytotoxic effector and memory T cells. They produce cytokines and other cytotoxic molecules to kill cells that express specific antigen [80]. Studies have revealed that during pregnancy, decidual natural killer cells, which are mainly cytokines and chemokines producers, become cytotoxic effectors upon their exposure to CMV [73]. CMV have been found to induce local macrophage and T-cell infiltration at the site of infection in vivo [81], disrupt trophoblast invasion and proliferation [82] and alter immune function [74,83].

The first known detailed report of the association between congenital CMV and autistic symptoms was published in 1978 by Stubbs [84], followed by several other cases in the years after [13,85]. Congenital CMV infection was suggested by an antibody response to the virus, positive virus culture from the urine, impaired hearing, and inflammation of the retina. Prenatal or congenital CMV infection is one of the leading cause of sensorineural hearing loss [86,87] and other neurological disabilities [88,89]. Earlier case studies have also reported that children with congenital CMV infection manifested typical autistic characteristics including failure to develop good interpersonal relationship, poor eye-contact, delayed use of language and nonthematic use of objects. There were several cases of severely disabled children with autism and congenital CMV infection in 1990. Maternal infection was suspected as indicated serologically by high CMV-IgM titre [90]. In another study, positive serum CMV-specific IgM antibodies and also CMV-DNA in the urine were also observed in children that developed typical autistic disorder [14]. Findings of the cranial magnetic resonance imaging (MRI) revealed an abnormally intense area in the periventricular white matter, which suggested disturbed myelination. Sweeten, et al. [91] briefly described the possible role of congenital and perinatal CMV infection in triggering an altered immune response or autoimmune process. The virus or the ensuing immune response could disrupt the development of brain region or structures leading to the syndrome of autism.

Congenital CMV infection can be diagnosed early by CMV DNA screening in cord blood using real time polymerase chain reaction (RT-PCR) instead of using the gold standard method of detecting CMV in urine within the first two weeks of life [92]. Newborns positive for CMV DNA showed no abnormalities at birth but head MRI at 12 months showed lesions in white matters from temporal to posterior regions. In children with developmental delay, developmental tests conducted around the same time as MRI have shown an association with congenital CMV infection. ASD has been reported in children with congenital CMV infection who were previously asymptomatic at birth. Kawatani, et al. [93], reported that a four-year-old boy who was retrospectively diagnosed with CMV infection was born healthy but developed autistic features at the age of two. Positive CMV DNA from the umbilical cord and neurologic abnormalities revealed on brain CT were associated with ASD. Teratogenic effects of CMV infection was detected by neuroimaging in a cohort of children with neurological disability and cerebral cortical malformations [94]. In this study, the analysis of CMV DNA from dried blood spot samples using qualitative PCR method indicated 4 out of 26 children had congenital CMV infection. Two of them had severe disabilities with intellectual disability, ASD, cerebral palsy, epilepsy and deafness.

In another study by Engman, et al. [95], the prevalence of congenital CMV infection was evaluated in a representative sample of children with ASD. One of the 33 children with autistic disorder and intellectual disabilities had congenital CMV infection, corresponding to the prevalence of 0.2% in the general Swedish newborn population. Sakamoto, et al. [96] suggested involvement of congenital CMV infection in a portion of children with ASD as the rate of CMV infection was higher than the incidence of congenital CMV infection in Nagasaki, Japan. CMV infection is considered an important congenital infection in developed countries. Though a substantial number of studies have been conducted on congenital CMV infection, its management is not yet well-defined. CMV infections can cause a variety of neural deficits including ASD, even in infants who are asymptomatic at birth. Thus, research on early treatment and long-term follow-up of susceptible infants are necessary. Vaccine development is a major public health priority but currently there is no licensed CMV vaccine available. However, progress towards this goal has been made in recent ongoing clinical trials [97].

### 4.3. Influenza and ASD

Influenza is an infectious respiratory disease caused by influenza A and influenza B viruses [98]. Both influenza A and influenza B viruses are enveloped negative-sense Orthomyxoviridae RNA viruses with a genome that contains 8 genomic segments. According to WHO, influenza viruses are prevalent worldwide, infecting 5 to 15% of the global population annually with over 3 million individuals developing severe disease, resulting in hundreds of thousands of deaths per year [99]. An influenza pandemic occurs every 10 to 50 years and is characterised by the introduction of a new strain of influenza A virus [98]. The severity of infection and the mortality rate is dependent on pre-existing immunity in humans. During the 2009 influenza A (H1N1) pandemic, pregnant women were among the most vulnerable to severe illnesses and death [100]. Death was frequently caused by severe influenza-associated lower respiratory tract infection and acute respiratory distress syndrome (ARDS). The occurrence of ARDS is mainly contributed by high viral load or high levels of inflammation in the lower airways. Young children, especially those who were born prematurely and pregnant women may develop serious complications when infected with influenza virus [101].

The host response to influenza virus could offer protection by limiting viral infection within the lung. These factors include alveolar epithelial cells, cells of the immune system (macrophages, T cells, B cells, and neutrophils), cytokines, chemokines, antibodies, and surfactant proteins. These viral and host factors promote the generation of inflammation in the lung, which attributes to severe influenza-like illness [101]. From animal studies and clinical reports, pregnancy has been found to increase the severity of influenza virus infection, and thus impairment of maternal and offspring health and recovery [102,103,104]. The adverse effects of influenza virus on the foetus during pregnancy are not directly mediated by infection of placenta and foetus, but indirectly by hormonal signalling dysregulation, inflammation or immune system activation against placental and/or foetal tissue [105]. In a mouse pregnancy model, influenza virus was found to disrupt the delicate and interconnected cytokine and hormonal signalling pathways that responded to respiratory pathogens.

Like other common viral pathogens such as rubella and cytomegalovirus, seasonal and pandemic influenza infections during pregnancy have also been associated with adverse neurodevelopmental outcomes such as ASD and schizophrenia. The association between influenza infection and psychosis have been documented as “psychoses of influenza” during multiple pandemics since the eighteenth century [45]. Infection with influenza viruses during pregnancy increased the risk of complications including preterm labour, preterm delivery and birth defects [106]. Although literature concerning gestational maternal influenza virus infection and risk of ASD is inconclusive, some of epidemiological studies suggest that it may increase the risk of ASD in offspring. The link between influenza infection and human neurodevelopmental disorders has been established from animal models of gestational influenza [15] and prenatal serological testing of schizophrenia [107].

Several evidences gathered from self-reported data or medical records have linked maternal influenza virus infection to ASD. A population-based cohort study reported almost a twofold increased risk of autism after self-reported gestational influenza virus infection [108]. The study also found an approximately threefold increase in risk of autism if the mother had suffered febrile episodes lasting more than one week, or prolonged episodes of fever during pregnancy. In an earlier case-control study, Deykin and Macmahon [11] determined that nearly 8% of mothers whose pregnancy have had or been exposed to influenza resulted in autistic children. However, a recent cohort study by Zerbo, et al. [109] reported that maternal influenza infection at any time during pregnancy was not associated with increased risk of ASD. This finding is similar to previous study by Dassa, et al. [110] who reported neither ASD nor developmental delay was associated with gestational influenza infection. Zerbo, et al. [111] found no association between ASD and influenza infection, however, mothers with children with autism were more likely to report fever during pregnancy compared to mothers with children without autism. Fever is one of the early clinical symptoms of influenza infection, accompanied by the respiratory symptoms [98]. Hyperthermia and fever during pregnancy have been suggested to cause a wide range of functional and structural neural tube defects [112,113] and may be the initiating factors of some neurological conditions including ASD, schizophrenia, and cerebral palsy.

Since prenatal influenza virus infection was associated with an increased risk of schizophrenia and autism, the Advisory Committee on Immunisation Practices recommends that pregnant women should receive inactivated influenza vaccine at any stage of gestation [114]. Several studies have been carried out to demonstrate maternal immune activation in a mouse model of pregnancy via exposure to viruses or lipopolysaccharides (LPS). Foetal inflammation elicited by maternal injections of LPS has notable detrimental effects on brain development, underlying the neurobehavioural deficits reported in humans and animals that were exposed to prenatal insults. Pre-treatment with influenza vaccination has been shown to promote neurogenesis both in pregnant mice and in their offspring [115,116]. In addition, influenza vaccination pre-treatment in an LPS-induced maternal immune activation mouse model has been found to promote exploratory behaviours in offspring and block subsequent LPS challenge from causing spatial cognitive impairments [115]. These findings were also accompanied by a decrease in proinflammatory cytokines induced by LPS treatment. These studies of influenza vaccine pre-treatment in an LPS-induced maternal immune activation model suggested its potential in exerting protective effects against autism-like behaviours.

### 4.4. Zika Virus and ASD

Zika virus (ZIKV) is one of the emerging viruses that also possess a significant threat to human health globally. It is an anthropod-borne virus or arbovirus (genus: Flavivirus, family: Flaviviradae) and usually transmitted by the bite of infected Aedes mosquitoes. The nonspecific clinical presentation of Zika fever can be misdiagnosed as other infectious diseases especially those due to arboviruses such as dengue and chikungunya [117]. ZIKV infection may cause mild influenza-like illness to serious manifestations and there is no specific anti-viral treatment for this virus. Previous reports suggested that ZIKV might be susceptible to existing anti-viral drugs that may prevent disruption to the fetal developing nervous system [118]. No vaccines are available for ZIKV infection in the market but there are currently about 45 candidates in trial phase [119].

The identification of ZIKV outbreak in early 2015 in a region of Brazil has led to the discovery that perinatal Zika exposure may cause congenital Zika syndrome. This was based on reports on the increased number of children born with severe microcephaly in the same geographic area in Brazil [120]. In 2016, CDC has updated its interim guideline caring for pregnant woman during ZIKV outbreak for US healthcare providers [121]. Studies have demonstrated that the Brazilian ZIKV strain is able to cross placental barrier, infect progenitor cortical cells and promoted cell death by inducing apoptosis and autophagy [122]. This virus reached the fetal neural cells through infection of cytotrophoblasts or transmigration of infected primary human placental macrophages [123]. Previous reports associated the presence of ZIKV specific IgM with microchephaly in neonates. ZIKV may have caused microcephaly by killing neural precursor cells and other brain cells [124]. Congenital Zika syndrome is associated with severe developmental delay and cerebral palsy after one year of age. Although most children born without microcephaly may have typical development, some may be at risk for neurodevelopment impairments, especially in the language domain [125].

A longitudinal infant cohort study with RT-PCR confirmed maternal ZIKV infection in pregnancy during the Rio de Janeiro epidemic of 2015–2016. Nielsen-Saines et al. [126] 2020 reported three children that were initially normal at birth developed ASD during the second year of life. Meanwhile, among the 18 children with very low average performance, 6 were microcephalic and developed ASD. In a case series study by Abtibol-Bernardino et al. [127], the neurological assessment of 26 non-microcephalic children who had intrauterine exposure to Zika virus showed that majority of the children obtained satisfactory performance in neurodevelopment, while among five children that had severe neurological disorders, two of them were presented with autism. Neuroimmune modulation has been suggested to play a role in the genesis of autism in infants exposed to congenital ZIKV infection. Though it is not well understood how the immune system response to ZIKV infection is regulated and how it is associated to ASD, it is recognised that the inflammatory response to ZIKV infection and the brain damage caused by the virus in affected infants could favor the development of neurodevelopmental disorders such as ASD [123]. This is based on the fact that pro-inflammatory cytokines such as IL-6 and TNF-α are released at high levels in response to viral infection and hence predisposed the occurrence of the disorder.

In another study, a population-based mother–child cohort study of women who were pregnant during the 2016 ZIKV outbreak in America has found that 15.3% of toddler exposed to ZIKV in utero to have abnormal neurodevelopment findings at 24 months of age [128]. The neurodevelopment assessments include the five dimension of general development (communication, fine motor, gross motor, problem solving, and personal–social skills) and the modified checklist for autism on toddlers for behaviour. However the study has found that there were no differences between ZIKV exposed and ZIKV un-exposed toddlers for behaviour disorder screening risk nor for language acquisition. However, the authors suggested that it is important to continue the neurobehavioural assessment into early childhood. In addition, the children who was born without microcephaly but were exposed to ZIKV infection in intrauterine life, careful follow-up and monitoring of their neurodevelopment are still necessary [125].

### 4.5. COVID-19 and Possible Risk of ASD

In early 2020, WHO declared COVID-19 as a public health emergency of international concern. The novel coronavirus that caused COVID-19 has posed a severe threat to the entire world. In February 2020, The International Committee on Taxonomy of Viruses (ICTV) has announced the official name for the virus responsible for COVID-19 as “Severe Acute Respiratory Syndrome—Coronavirus-2 (SARS-CoV-2)”. SARS-CoV-2 is a beta coronavirus that is strictly related to a virus (MERS-CoV) that caused Middle East respiratory syndrome (MERS) and virus (SARS-CoV) that caused severe acute respiratory syndrome (SARS). Similar cytokine spike can be observed during SARS-CoV, MERS-CoV, and SARS-CoV-2 infection. The severity of these viral infections were positively correlated with the levels of IL-17 and other T-helpers 17 cell-related proinflammatory cytokines, such as IL-1, IL-6, IL-15, TNF, and IFN-γ [129,130].

Coronavirus primarily targets the human respiratory system but it also has neuro-invasive capabilities by spreading from the respiratory tract to the CNS. Upon nasal infection, the virus enters the CNS through the olfactory bulb and can eventually lead to inflammation and demyelination [131]. The neuro-invasive abilities of the coronavirus was evidenced by COVID-19 patients presenting with febrile seizures, encephalitis, convulsions, and altered mental status [132]. Some patients showed non-specific neurological symptoms such as headache, dizziness, and confusion. Generally, after two weeks of incubations period, COVID-19 patient will experience a cytokine storm in an attempt to clear the infectious agent. Cytokine storm is a concern for pregnant woman as the imbalance of cytokine expressions may expose the foetus to a higher chance of having ASD. In an in vitro study, the SARS-CoV replication in Caco-2 cells induced high-level expressions of IL-6 and Regulated upon Activation, Normal T Cell Expressed and Presumably Secreted (RANTES) compared to cells infected with Influenza A and Human parainfluenza-2 virus [133]. Excessive induction of the inflammatory cytokines and dysregulation of cytokines signaling were associated with severe inflammation in SARS. Analysis of serum cytokines in patients with SARS showed that IL-6 and IFN-γ, which promote inflammation by inducing injury, were significantly increased. This cytokine change was associated with disease severity and might be involved in the immunopathological damage seen in SARS patient [134,135].

Although much attention has been given to older adults population for being the high-risk group susceptible to life threatening conditions associated to SARS-CoV-2 infection, pregnant women should also be regarded as high risk population that needs as much attention for prevention and management of this infectious disease [136]. A recent systematic review showed no evidence on the presence of SARS-CoV-2 in breast milk of pregnant women with COVID-19, indicating lack of evidence for vertical transmission of SARS-CoV-2 [137]. In a retrospective cohort study, the presence of SARS-CoV-2 by RT-PCR were not detected in amniotic fluid, placenta, neonatal throat, and anal swab as well as in breast milk samples of pregnant women with COVID-19 [138]. Nonetheless, dysregulation of inflammation process may have important implications in pregnant women with SARS-CoV-2. In addition, studies evaluating the effects of inflammation dysregulation on the pathophysiology and genomic function of the placenta and the potential short- and long-term effects on children development are yet to be explored [136]. Potential risk for neurodevelopmental disorders in neonates are explainable in the light of the recent findings that pregnant women with SARS-CoV-2 infection had higher levels of IL-6 [138].

A recent prospective longitudinal study found that maternal IL-6 was inversely associated with offspring cognitive development at 12-months of age. Structural connectivity of fronto-limbic circuitry is critical for socioemotional and cognitive development. Changes in fronto-limbic white matter detected by MRI scans were associated with maternal IL-6 concentration during pregnancy [139]. This finding strengthens the assumption that maternal inflammation may adversely affect intrauterine condition, exposing the foetus to neurodevelopmental disorders. Hence, the assessment of inflammatory levels in pregnant women with COVID-19 and longitudinal evaluation of offspring neurodevelopmental outcomes are important in view of the current COVID-19 pandemic. Since we do not yet fully understand the entire pathophysiology of COVID-19, recent evidences can support the hypothesis of ASD being a risk factor but further studies are necessary to confirm this correlation.

## 5. Summary

In this paper, we reviewed the possible association between maternal infection during pregnancy and ASD. Various risk factors can disrupt fetal development due to the fact that neurological development is the product of a multifactorial process that depends on the interaction between genetic and environmental aspects. Infectious agents that can cross the placental barrier and enter the fetal bloodstream can cause direct damage by promoting cytotoxic effect, mitotic inhibition or any events that lead to vascular disruption. An infectious agent could also induce an aggressive reparative response that lead to increase lesional area and intracranial calcifications [140]. It is known that infectious agents such as rubella, cytomegalovirus and herpes viruses can alter brain development and caused impairments such as brain calcifications, microcephaly and other neurodevelopment disorders. Altogether, compiled evidence suggest that these complications reported during pregnancy are associated with neurodevelopment disorders particularly ASD.

However, there are studies that indicated no association between maternal infections during pregnancy and risk of ASD [141]. These reports are in contrast to previous reports indicating that rubella, cytomegalovirus, and influenza increased risk of ASD. Reasons behind these observations could be due to diagnoses of those viruses were not documented in medical records on the time of the study, thus contributing to no association findings. On the other hand, there were possibilities that the studies, which were based on self-reported infection, may contribute to over-counting or under-counting of infection cases. These could be related to limited access to health care and care-seeking behaviour in the patients [141]. In addition, some studies were observational studies, which are prone to unclear risk of bias in the selection of subjects and blinding of outcome assessments across studies. Studies with small sample groups, heterogenous methods and study protocols may yield controversial/variable results. Hence, further studies should consider the potential confounders and include more participants to strengthen statistical power. High phenotypic heterogeneity for instance can be minimised by classification of study subjects into clinical subgroups based on symptom clusters.

Nonetheless, reported studies indicated that the inflammatory process is a common key factor in response to the infections. Better understanding of the normal neurodevelopment and its comparison with neuropathogenesis in emerging viral infection is required for the identification of virus target cells and its possible neurodevelopmental damages. Further researches are warranted to elucidate the precise mechanisms of how immune activation may cause neurobiological and neurochemical abnormalities relevant to ASD. It is also important to evaluate the long-term effect of maternal viral infection and its correlation with ASD. This knowledge is critical for the establishment of modulatory preventive measures and treatment of ASD.

## 6. Conclusions

In conclusion, maternal immune activation involving cytokine network dysfunction may be the potential pathogenic process that link viral infections with the risk of ASD in children. Research must be continued to further understand the role of viral infection in the aetiology of ASD.

## References

[1] American Psychiatric Association (2000). Diagnostic and Statistical Manual of Mental Disorders.

[2] Newschaffer C.J., Croen L.A., Daniels J., Giarelli E., Grether J.K., Levy S.E., Mandell D.S., Miller L.A., Pinto-Martin J., Reaven J. (2007). The epidemiology of autism spectrum disorders. Annu. Rev. Public Health.

[3] Gillberg C., Wing L. (1999). Autism: Not an extremely rare disorder. Acta Psychiatr. Scand..

[4] Wilczynski S., Pollack E. (2011). Evidence-Based Pratice and Autism in the Schools: A Guide to Providing Appropriate Interventions to Studentes with Autism Spectrum Disorders.

[5] Baxter A.J., Brugha T., Erskine H.E., Scheurer R.W., Vos T., Scott J.G. (2015). The epidemiology and global burden of autism spectrum disorders. Psychol. Med..

[6] Christensen D.L., Braun K.V.N., Baio J., Bilder D., Charles J., Constantino J.N., Daniels J., Durkin M.S., Fitzgerald R.T., Kurzius-Spencer M. (2018). Prevalence and Characteristics of Autism Spectrum Disorder Among Children Aged 8 Years—Autism and Developmental Disabilities Monitoring Network, 11 Sites, United States, 2012. MMWR Surveill. Summ..

[7] Ornoy A., Weinstein-Fudim L., Ergaz Z. (2016). Genetic syndromes, maternal diseases and antenatal factors associated with autism spectrum disorders (ASD). Front. Neurosci..

[8] Libbey J.E., Sweeten T.L., McMahon W.M., Fujinami R.S. (2005). Autistic disorder and viral infections. J. Neurovirol..

[9] Meltzer A., Van de Water J. (2017). The role of the immune system in autism spectrum disorder. Neuropsychopharmacology.

[10] Chess S., Fernandez P., Korn S. (1978). Behavioral consequences of congenital rubella. J. Pediatrics.

[11] Deykin E.Y., Macmahon B. (1979). Viral exposure and autism. Am. J. Epidemiol..

[12] Lintas C., Altieri L., Lombardi F., Sacco R., Persico A.M. (2010). Association of autism with polyomavirus infection in postmortem brains. J. Neurovirol..

[13] Markowitz P.I. (1983). Autism in a child with congenital cytomegalovirus infection. J. Autism Dev. Disord..

[14] Yamashita Y., Fujimoto C., Nakajima E., Isagai T., Matsuishi T. (2003). Possible association between congenital cytomegalovirus infection and autistic disorder. J. Autism Dev. Disord..

[15] Shi L., Fatemi S.H., Sidwell R.W., Patterson P.H. (2003). Maternal influenza infection causes marked behavioral and pharmacological changes in the offspring. J. Neurosci..

[16] Patterson P.H. (2011). Maternal infection and immune involvement in autism. Trends Mol. Med..

[17] Asp L., Beraki S., Kristensson K., Ögren S.O., Karlsson H. (2009). Neonatal infection with neurotropic influenza A virus affects working memory and expression of type III Nrg1 in adult mice. Brain Behav. Immun..

[18] Patterson P.H. (2002). Maternal infection: Window on neuroimmune interactions in fetal brain development and mental illness. Curr. Opin. Neurobiol..

[19] Amor S., Puentes F., Baker D., Van Der Valk P. (2010). Inflammation in neurodegenerative diseases. Immunology.

[20] Ghoshal A., Das S., Ghosh S., Mishra M.K., Sharma V., Koli P., Sen E., Basu A. (2007). Proinflammatory mediators released by activated microglia induces neuronal death in Japanese encephalitis. Glia.

[21] Zhou L., Miranda-Saksena M., Saksena N.K. (2013). Viruses and neurodegeneration. Virol. J..

[22] Karim S., Mirza Z., A Kamal M., M Abuzenadah A., I Azhar E., H Al-Qahtani M., A Damanhouri G., Ahmad F., H Gan S., S Sohrab S. (2014). The role of viruses in neurodegenerative and neurobehavioral diseases. CNS Neurol. Disord. Drug Targets (Former. Curr. Drug Targets-CNS Neurol. Disord.).

[23] Kourtis A.P., Read J.S., Jamieson D.J. (2014). Pregnancy and infection. N. Engl. J. Med..

[24] Medearis D.N. (1964). Viral infections during pregnancy and abnormal human development. Am. J. Obstet. Gynecol..

[25] Cordeiro C.N., Tsimis M., Burd I. (2015). Infections and brain development. Obstet. Gynecol. Surv..

[26] Waldorf K.M.A., McAdams R.M. (2013). Influence of infection during pregnancy on fetal development. Reproduction.

[27] Johnson R.T. (1994). Infections during pregnancy. Adv. Neurol..

[28] Lee B.K., Magnusson C., Gardner R.M., Blomström Å., Newschaffer C.J., Burstyn I., Karlsson H., Dalman C. (2015). Maternal hospitalization with infection during pregnancy and risk of autism spectrum disorders. Brain Behav. Immun..

[29] Meyer U., Feldon J., Dammann O. (2011). Schizophrenia and autism: Both shared and disorder-specific pathogenesis via perinatal inflammation?. Pediatric Res..

[30] Dammann O., Leviton A. (2000). Role of the fetus in perinatal infection and neonatal brain damage. Curr. Opin. Pediatrics.

[31] Zhu J., Paul W.E. (2008). CD4 T cells: Fates, functions, and faults. Blood J. Am. Soc. Hematol..

[32] Choi G.B., Yim Y.S., Wong H., Kim S., Kim H., Kim S.V., Hoeffer C.A., Littman D.R., Huh J.R. (2016). The maternal interleukin-17a pathway in mice promotes autism-like phenotypes in offspring. Science.

[33] Smith S.E., Li J., Garbett K., Mirnics K., Patterson P.H. (2007). Maternal immune activation alters fetal brain development through interleukin-6. J. Neurosci..

[34] Eftekharian M.M., Ghafouri-Fard S., Noroozi R., Omrani M.D., Arsang-Jang S., Ganji M., Gharzi V., Noroozi H., Komaki A., Mazdeh M. (2018). Cytokine profile in autistic patients. Cytokine.

[35] Rudolph M.D., Graham A.M., Feczko E., Miranda-Dominguez O., Rasmussen J.M., Nardos R., Entringer S., Wadhwa P.D., Buss C., Fair D.A. (2018). Maternal IL-6 during pregnancy can be estimated from newborn brain connectivity and predicts future working memory in offspring. Nat. Neurosci..

[36] Estes M.L., McAllister A.K. (2016). Maternal immune activation: Implications for neuropsychiatric disorders. Science.

[37] Vargas D.L., Nascimbene C., Krishnan C., Zimmerman A.W., Pardo C.A. (2005). Neuroglial activation and neuroinflammation in the brain of patients with autism. Ann. Neurol. Off. J. Am. Neurol. Assoc. Child Neurol. Soc..

[38] Chez M.G., Dowling T., Patel P.B., Khanna P., Kominsky M. (2007). Elevation of tumor necrosis factor-alpha in cerebrospinal fluid of autistic children. Pediatric Neurol..

[39] Morgan J.T., Chana G., Pardo C.A., Achim C., Semendeferi K., Buckwalter J., Courchesne E., Everall I.P. (2010). Microglial activation and increased microglial density observed in the dorsolateral prefrontal cortex in autism. Biol. Psychiatry.

[40] Careaga M., Van de Water J., Ashwood P. (2010). Immune dysfunction in autism: A pathway to treatment. Neurotherapeutics.

[41] Ashwood P., Krakowiak P., Hertz-Picciotto I., Hansen R., Pessah I.N., Van de Water J. (2011). Associations of impaired behaviors with elevated plasma chemokines in autism spectrum disorders. J. Neuroimmunol..

[42] Ashwood P., Krakowiak P., Hertz-Picciotto I., Hansen R., Pessah I., Van de Water J. (2011). Elevated plasma cytokines in autism spectrum disorders provide evidence of immune dysfunction and are associated with impaired behavioral outcome. Brain Behav. Immun..

[43] AL-Ayadhi L.Y., Mostafa G.A. (2012). Elevated serum levels of interleukin-17A in children with autism. J. Neuroinflamm..

[44] Arad M., Piontkewitz Y., Albelda N., Shaashua L., Weiner I. (2017). Immune activation in lactating dams alters sucklings’ brain cytokines and produces non-overlapping behavioral deficits in adult female and male offspring: A novel neurodevelopmental model of sex-specific psychopathology. Brain Behav. Immun..

[45] Kępińska A.P., Iyegbe C.O., Vernon A.C., Yolken R., Murray R.M., Pollak T.A. (2020). Schizophrenia and influenza at the centenary of the 1918-1919 Spanish influenza pandemic: Mechanisms of psychosis risk. Front. Psychiatry.

[46] Estes M.L., McAllister A.K. (2015). Immune mediators in the brain and peripheral tissues in autism spectrum disorder. Nat. Rev. Neurosci..

[47] Depino A.M. (2013). Peripheral and central inflammation in autism spectrum disorders. Mol. Cell. Neurosci..

[48] Edlich R., Winters K.L., Long W.B. (2005). Rubella and congenital rubella (German measles). J. Long-Term Eff. Med Implant..

[49] Mawson A.R., Croft A.M. (2019). Rubella virus infection, the Congenital Rubella Syndrome, and the link to autism. Int. J. Environ. Res. Public Health.

[50] Miller E., Cradock-Watson J., Pollock T. (1982). Consequences of confirmed maternal rubella at successive stages of pregnancy. Lancet.

[51] Cooper L.Z., Krugman S. (1967). Clinical manifestations of postnatal and congenital rubella. Arch. Ophthalmol..

[52] Silasi M., Cardenas I., Kwon J.Y., Racicot K., Aldo P., Mor G. (2015). Viral infections during pregnancy. Am. J. Reprod. Immunol..

[53] Sever J.L., Nelson K.B., Gilkeson M.R. (1965). Rubella Epidemic, 1964: Effect on 6,000 Pregnancies: I. Preliminary Clinical and Laboratory Findings Through the Neonatal Period: A Report from the Collaborative Study on Cerebral Palsy. Am. J. Dis. Child..

[54] Gregg N.M. (1941). Congenital cataract following German measles in the mother. Problems of Birth Defects.

[55] Chess S. (1971). Autism in children with congenital rubella. J. Autism Child. Schizophr..

[56] Chess S. (1977). Follow-up report on autism in congenital rubella. J. Autism Child. Schizophr..

[57] Stubbs E.G. (1976). Autistic children exhibit undetectable hemagglutination-inhibition antibody titers despite previous rubella vaccination. J. Autism Child. Schizophr..

[58] Banatvala J.E., Brown D.W. (2004). Rubella. Lancet.

[59] O’Donnell N. (1991). A Report on a Survey of Late Emerging Manifestations of Congenital Rubella Syndrome.

[60] Hwang S.-J., Chen Y.-S. (2010). Congenital rubella syndrome with autistic disorder. J. Chin. Med. Assoc..

[61] Hofmann J., Pletz M., Liebert U. (1999). Rubella virus-induced cytopathic effect in vitro is caused by apoptosis. J. Gen. Virol..

[62] Pugachev K.V., Frey T.K. (1998). Rubella virus induces apoptosis in culture cells. Virology.

[63] Carvill S., Marston G. (2002). People with intellectual disability, sensory impairments and behaviour disorder: A case series. J. Intellect. Disabil. Res..

[64] Assumpção F.B., Kuczynski E. (2002). Autism, bipolar disorder and mental retardation in a male adolescent with congenital rubella: Case report. Arq. Neuro-Psiquiatr..

[65] Toizumi M., Nguyen G.T.H., Motomura H., Nguyen T.H., Pham E., Kaneko K.-I., Uematsu M., Nguyen H.A.T., Dang D.A., Hashizume M. (2017). Sensory defects and developmental delay among children with congenital rubella syndrome. Sci. Rep..

[66] Duester G. (2008). Retinoic acid synthesis and signaling during early organogenesis. Cell.

[67] Berger B.E., Navar-Boggan A.M., Omer S.B. (2011). Congenital rubella syndrome and autism spectrum disorder prevented by rubella vaccination-United States, 2001-2010. BMC Public Health.

[68] Hutton J. (2016). Does rubella cause autism: A 2015 reappraisal?. Front. Hum. Neurosci..

[69] Dinede G., Wondimagegnehu A., Enquselassie F. (2019). Rubella outbreak in the school children, Addis Ababa, Ethiopia: February–April 2018. BMC Infect. Dis..

[70] Weller T.H. (1971). The cytomegaloviruses: Ubiquitous agents with protean clinical manifestations. N. Engl. J. Med..

[71] Landolfo S., Gariglio M., Gribaudo G., Lembo D. (2003). The human cytomegalovirus. Pharmacol. Ther..

[72] Zhang X.-Y., Fang F. (2019). Congenital human cytomegalovirus infection and neurologic diseases in newborns. Chin. Med. J..

[73] Siewiera J., El Costa H., Tabiasco J., Berrebi A., Cartron G., Bouteiller P., Jabrane-Ferrat N. (2013). Human cytomegalovirus infection elicits new decidual natural killer cell effector functions. PLoS Pathog..

[74] Pizzato N., Garmy-Susini B., Le Bouteiller P., Lenfant F. (2003). Down-regulation of HLA-G1 cell surface expression in human cytomegalovirus infected cells. Am. J. Reprod. Immunol..

[75] Cannon M.J., Davis K.F. (2005). Washing our hands of the congenital cytomegalovirus disease epidemic. BMC Public Health.

[76] Boppana S.B., Fowler K.B., Pass R.F., Rivera L.B., Bradford R.D., Lakeman F.D., Britt W.J. (2005). Congenital cytomegalovirus infection: Association between virus burden in infancy and hearing loss. J. Pediatrics.

[77] Traylen C.M., Patel H.R., Fondaw W., Mahatme S., Williams J.F., Walker L.R., Dyson O.F., Arce S., Akula S.M. (2011). Virus reactivation: A panoramic view in human infections. Future Virol..

[78] Slawinski B.L., Talge N., Ingersoll B., Smith A., Glazier A., Kerver J., Paneth N., Racicot K. (2018). Maternal cytomegalovirus sero-positivity and autism symptoms in children. Am. J. Reprod. Immunol..

[79] Cicin-Sain L., Brien J.D., Uhrlaub J.L., Drabig A., Marandu T.F., Nikolich-Zugich J. (2012). Cytomegalovirus infection impairs immune responses and accentuates T-cell pool changes observed in mice with aging. PLoS Pathog..

[80] Lei J., Xie L., Zhao H., Gard C., Clemens J.L., McLane M.W., Feller M.C., Ozen M., Novak C., Alshehri W. (2018). Maternal CD 8+ T-cell depletion alleviates intrauterine inflammation-induced perinatal brain injury. Am. J. Reprod. Immunol..

[81] Aronoff D.M., Correa H., Rogers L.M., Arav-Boger R., Alcendor D.J. (2017). Placental pericytes and cytomegalovirus infectivity: Implications for HCMV placental pathology and congenital disease. Am. J. Reprod. Immunol..

[82] Liu T., Zheng X., Li Q., Chen J., Yin Z., Xiao J., Zhang D., Li W., Qiao Y., Chen S. (2015). Role of human cytomegalovirus in the proliferation and invasion of extravillous cytotrophoblasts isolated from early placentae. Int. J. Clin. Exp. Med..

[83] Chou D., Ma Y., Zhang J., McGrath C., Parry S. (2006). Cytomegalovirus infection of trophoblast cells elicits an inflammatory response: A possible mechanism of placental dysfunction. Am. J. Obstet. Gynecol..

[84] Stubbs E.G. (1978). Autistic symptoms in a child with congenital cytomegaloviras infection. J. Autism Child. Schizophr..

[85] Stubbs E.G., Ash E., Williams C.P. (1984). Autism and congenital cytomegalovirus. J. Autism Dev. Disord..

[86] Grosse S.D., Ross D.S., Dollard S.C. (2008). Congenital cytomegalovirus (CMV) infection as a cause of permanent bilateral hearing loss: A quantitative assessment. J. Clin. Virol..

[87] Fowler K.B., Dahle A.J., Boppana S.B., Pass R.F. (1999). Newborn hearing screening: Will children with hearing loss caused by congenital cytomegalovirus infection be missed?. J. Pediatrics.

[88] Dollard S.C., Grosse S.D., Ross D.S. (2007). New estimates of the prevalence of neurological and sensory sequelae and mortality associated with congenital cytomegalovirus infection. Rev. Med. Virol..

[89] Townsend C.L., Forsgren M., Ahlfors K., Ivarsson S.-A., Tookey P.A., Peckham C.S. (2013). Long-term outcomes of congenital cytomegalovirus infection in Sweden and the United Kingdom. Clin. Infect. Dis..

[90] Ivarsson S., Bjerre I., Vegfors P., Ahlfors K. (1990). Autism as one of several disabilities in two children with congenital cytomegalovirus infection. Neuropediatrics.

[91] Sweeten T.L., Posey D.J., McDougle C.J. (2004). Brief report: Autistic disorder in three children with cytomegalovirus infection. J. Autism Dev. Disord..

[92] Endo T., Goto K., Ito K., Sugiura T., Terabe K., Cho S., Nishiyama M., Sugiyama K., Togari H. (2009). Detection of congenital cytomegalovirus infection using umbilical cord blood samples in a screening survey. J. Med. Virol..

[93] Kawatani M., Nakai A., Okuno T., Kobata R., Moriuchi M., Moriuchi H., Tsukahara H., Mayumi M. (2010). Detection of cytomegalovirus in preserved umbilical cord from a boy with autistic disorder. Pediatrics Int..

[94] Engman M.L., Lewensohn-Fuchs I., Mosskin M., Malm G. (2010). Congenital cytomegalovirus infection: The impact of cerebral cortical malformations. Acta Paediatr..

[95] Engman M.L., Sundin M., Miniscalco C., Westerlund J., Lewensohn-Fuchs I., Gillberg C., Fernell E. (2015). Prenatal acquired cytomegalovirus infection should be considered in children with autism. Acta Paediatr..

[96] Sakamoto A., Moriuchi H., Matsuzaki J., Motoyama K., Moriuchi M. (2015). Retrospective diagnosis of congenital cytomegalovirus infection in children with autism spectrum disorder but no other major neurologic deficit. Brain Dev..

[97] Anderholm K.M., Bierle C.J., Schleiss M.R. (2016). Cytomegalovirus Vaccines: Current Status and Future Prospects. Drugs.

[98] Krammer F., Smith G.J.D., Fouchier R.A.M., Peiris M., Kedzierska K., Doherty P.C., Palese P., Shaw M.L., Treanor J., Webster R.G. (2018). Influenza. Nat. Rev. Dis. Primers.

[99] Doyon-Plourde P., Fakih I., Tadount F., Fortin É., Quach C. (2019). Impact of influenza vaccination on healthcare utilization—A systematic review. Vaccine.

[100] Louie J.K., Acosta M., Jamieson D.J., Honein M.A. (2010). Severe 2009 H1N1 influenza in pregnant and postpartum women in California. N. Engl. J. Med..

[101] Misra R.S., Nayak J.L. (2019). The Importance of Vaccinating Children and Pregnant Women against Influenza Virus Infection. Pathogens.

[102] Chan K.-H., Zhang A.J., To K.K., Chan C.C., Poon V.K., Guo K., Ng F., Zhang Q.-W., Cheung A.N., Lau C.C. (2010). Wild type and mutant 2009 pandemic influenza A (H1N1) viruses cause more severe disease and higher mortality in pregnant BALB/c mice. PLoS ONE.

[103] Kim J.C., Kim H.M., Kang Y.M., Ku K.B., Park E.H., Yum J., Kim J.A., Kang Y.K., Lee J.S., Kim H.S. (2014). Severe pathogenesis of influenza B virus in pregnant mice. Virology.

[104] Chan J.F.-W., To K.K.-W., Tse H., Lau C.C.-Y., Li I.W.-S., Hung I.F.-N., Chan K.-H., Cheng V.C.-C., Lai T.S.-T., Woo P.C.-Y. (2011). The lower serum immunoglobulin G2 level in severe cases than in mild cases of pandemic H1N1 2009 influenza is associated with cytokine dysregulation. Clin. Vaccine Immunol..

[105] Littauer E.Q., Esser E.S., Antao O.Q., Vassilieva E.V., Compans R.W., Skountzou I. (2017). H1N1 influenza virus infection results in adverse pregnancy outcomes by disrupting tissue-specific hormonal regulation. PLoS Pathog..

[106] Raj R.S., Bonney E.A., Phillippe M. (2014). Influenza, immune system, and pregnancy. Reprod. Sci..

[107] Brown A.S., Begg M.D., Gravenstein S., Schaefer C.A., Wyatt R.J., Bresnahan M., Babulas V.P., Susser E.S. (2004). Serologic evidence of prenatal influenza in the etiology of schizophrenia. Arch. Gen. Psychiatry.

[108] Atladóttir H., Henriksen T.B., Schendel D.E., Parner E.T. (2012). Autism after infection, febrile episodes, and antibiotic use during pregnancy: An exploratory study. Pediatrics.

[109] Zerbo O., Qian Y., Yoshida C., Fireman B.H., Klein N.P., Croen L.A. (2017). Association between influenza infection and vaccination during pregnancy and risk of autism spectrum disorder. JAMA Pediatrics.

[110] Dassa D., Takei N., Sham P., Murray R. (1995). No association between prenatal exposure to influenza and autism. Acta Psychiatr. Scand..

[111] Zerbo O., Iosif A.-M., Walker C., Ozonoff S., Hansen R.L., Hertz-Picciotto I. (2013). Is maternal influenza or fever during pregnancy associated with autism or developmental delays? Results from the CHARGE (CHildhood Autism Risks from Genetics and Environment) study. J. Autism Dev. Disord..

[112] Edwards M.J. (2006). Review: Hyperthermia and fever during pregnancy. Birth Defects Res. Part A Clin. Mol. Teratol..

[113] Moretti M.E., Bar-Oz B., Fried S., Koren G. (2005). Maternal hyperthermia and the risk for neural tube defects in offspring: Systematic review and meta-analysis. Epidemiology.

[114] Xia Y., Qi F., Zou J., Yao Z. (2014). Influenza A (H1N1) vaccination during early pregnancy transiently promotes hippocampal neurogenesis and working memory. Involvement of Th1/Th2 balance. Brain Res..

[115] Xia Y., Qi F., Zou J., Yang J., Yao Z. (2014). Influenza vaccination during early pregnancy contributes to neurogenesis and behavioral function in offspring. Brain Behav. Immun..

[116] Qi F., Yang J., Xia Y., Yuan Q., Guo K., Zou J., Yao Z. (2016). A (H1N1) vaccination recruits T lymphocytes to the choroid plexus for the promotion of hippocampal neurogenesis and working memory in pregnant mice. Brain Behav. Immun..

[117] Musso D., Gubler D.J. (2016). Zika Virus. Clin. Microbiol. Rev..

[118] Onorati M., Li Z., Liu F., Sousa A.M., Nakagawa N., Li M., Dell’Anno M.T., Gulden F.O., Pochareddy S., Tebbenkamp A.T. (2016). Zika virus disrupts phospho-TBK1 localization and mitosis in human neuroepithelial stem cells and radial glia. Cell Rep..

[119] Javed F., Manzoor K.N., Ali M., Haq I.U., Khan A.A., Zaib A., Manzoor S. (2018). Zika virus: What we need to know?. J. Basic Microbiol..

[120] Oliveira Melo A.S., Malinger G., Ximenes R., Szejnfeld P., Alves Sampaio S., Bispo de Filippis A. (2016). Zika virus intrauterine infection causes fetal brain abnormality and microcephaly: Tip of the iceberg?. Ultrasound Obstet. Gynecol..

[121] Oduyebo T., Petersen E.E., Rasmussen S.A., Mead P.S., Meaney-Delman D., Renquist C.M., Ellington S.R., Fischer M., Staples J.E., Powers A.M. (2016). Update: Interim guidelines for health care providers caring for pregnant women and women of reproductive age with possible Zika virus exposure—United States, 2016. Morb. Mortal. Wkly. Rep..

[122] Cugola F.R., Fernandes I.R., Russo F.B., Freitas B.C., Dias J.L.M., Guimarães K.P., Benazzato C., Almeida N., Pignatari G.C., Romero S. (2016). The Brazilian Zika virus strain causes birth defects in experimental models. Nature.

[123] Vianna P., Gomes J.A., Boquett J.A., Fraga L.R., Schuch J.B., Vianna F.S.L., Schuler-Faccini L. (2018). Zika Virus as a Possible Risk Factor for Autism Spectrum Disorder: Neuroimmunological Aspects. Neuroimmunomodulation.

[124] Zhu Z., Mesci P., Bernatchez J.A., Gimple R.C., Wang X., Schafer S.T., Wettersten H.I., Beck S., Clark A.E., Wu Q. (2020). Zika virus targets glioblastoma stem cells through a SOX2-integrin αvβ5 axis. Cell Stem Cell.

[125] Carvalho A., Sales H.F., Ventura P., Gnoatto-Medeiros M., Brites C., Lucena R. (2020). The neurodevelopmental spectrum of congenital Zika infection: A scoping review. Dev. Med. Child Neurol..

[126] Nielsen-Saines K., Brasil P., Kerin T., Vasconcelos Z., Gabaglia C.R., Damasceno L., Pone M., de Carvalho L.M.A., Pone S.M., Zin A.A. (2019). Delayed childhood neurodevelopment and neurosensory alterations in the second year of life in a prospective cohort of ZIKV-exposed children. Nat. Med..

[127] Abtibol-Bernardino M.R., de Almeida Peixoto L.d.F.A., de Oliveira G.A., de Almeida T.F., Rodrigues G.R.I., Otani R.H., Soares Chaves B.C., de Souza Rodrigues C., de Andrade A.B.C.A., de Fatima Redivo E. (2020). Neurological Findings in Children without Congenital Microcephaly Exposed to Zika Virus in Utero: A Case Series Study. Viruses.

[128] Grant R., Fléchelles O., Tressières B., Dialo M., Elenga N., Mediamolle N., Mallard A., Hebert J.-C., Lachaume N., Couchy E. (2021). In utero Zika virus exposure and neurodevelopment at 24 months in toddlers normocephalic at birth: A cohort study. BMC Med..

[129] Mahallawi W.H., Khabour O.F., Zhang Q., Makhdoum H.M., Suliman B.A. (2018). MERS-CoV infection in humans is associated with a pro-inflammatory Th1 and Th17 cytokine profile. Cytokine.

[130] Ye Q., Wang B., Mao J. (2020). Cytokine storm in COVID-19 and treatment. J. Infect..

[131] Bohmwald K., Galvez N., Ríos M., Kalergis A.M. (2018). Neurologic alterations due to respiratory virus infections. Front. Cell. Neurosci..

[132] Asadi-Pooya A.A., Simani L. (2020). Central nervous system manifestations of COVID-19: A systematic review. J. Neurol. Sci..

[133] Okabayashi T., Kariwa H., Yokota S.I., Iki S., Indoh T., Yokosawa N., Takashima I., Tsutsumi H., Fujii N. (2006). Cytokine regulation in SARS coronavirus infection compared to other respiratory virus infections. J. Med. Virol..

[134] Zhang Y., Li J., Zhan Y., Wu L., Yu X., Zhang W., Ye L., Xu S., Sun R., Wang Y. (2004). Analysis of serum cytokines in patients with severe acute respiratory syndrome. Infect. Immun..

[135] Huang K.J., Su I.J., Theron M., Wu Y.C., Lai S.K., Liu C.C., Lei H.Y. (2005). An interferon-γ-related cytokine storm in SARS patients. J. Med. Virol..

[136] Martins-Filho P.R., Tanajura D.M., Santos H.P., Santos V.S. (2020). COVID-19 during pregnancy: Potential risk for neurodevelopmental disorders in neonates?. Eur. J. Obstet. Gynecol. Reprod. Biol..

[137] Martins-Filho P.R., Santos V.S., Santos H.P. (2020). To breastfeed or not to breastfeed? Lack of evidence on the presence of SARS-CoV-2 in breastmilk of pregnant women with COVID-19. Rev. Panam. Salud Pública.

[138] Yin M., Zhang L., Deng G., Han C., Shen M., Sun H., Zeng F., Zhang W., Chen L., Luo Q. (2020). Severe acute respiratory syndrome coronavirus 2 (SARS-CoV-2) infection during pregnancy in China: A retrospective cohort study. MedRxiv.

[139] Rasmussen J.M., Graham A.M., Entringer S., Gilmore J.H., Styner M., Fair D.A., Wadhwa P.D., Buss C. (2019). Maternal Interleukin-6 concentration during pregnancy is associated with variation in frontolimbic white matter and cognitive development in early life. Neuroimage.

[140] Calado A.M., dos Anjos Pires M. (2018). An overview of teratology. Teratog. Test..

[141] Zerbo O., Qian Y., Yoshida C., Grether J.K., Van de Water J., Croen L.A. (2015). Maternal Infection During Pregnancy and Autism Spectrum Disorders. J. Autism Dev. Disord..

